# Developing functional relationships between waterlogging and cotton growth and physiology-towards waterlogging modeling

**DOI:** 10.3389/fpls.2023.1174682

**Published:** 2023-07-31

**Authors:** Sahila Beegum, Van Truong, Raju Bheemanahalli, David Brand, Vangimalla Reddy, Kambham Raja Reddy

**Affiliations:** ^1^ Adaptive Cropping System Laboratory, USDA-ARS, Beltsville, MD, United States; ^2^ Nebraska Water Center, Robert B. Daugherty Water for Food Global Institute, University of Nebraska, Lincoln, NE, United States; ^3^ Department of Plant and Soil Sciences, Mississippi State University, Mississippi State, MS, United States

**Keywords:** cotton, waterlogging, plant physiology, morphology, waterlogging stress response index, functional relationships, process-based models

## Abstract

Cotton crop is known to be poorly adapted to waterlogging, especially during the early growth stages. Developing functional relationships between crop growth and development parameters and the duration of waterlogging is essential to develop or improve existing cotton crop models for simulating the impact of waterlogging. However, there are only limited experimental studies conducted on cotton specifically aimed at developing the necessary functional relationships required for waterlogging modeling. Further research is needed to understand the effects of waterlogging on cotton crops and improve modeling capabilities in this area. The current study aimed to conduct waterlogging experiments and develop functional relationships between waterlogging and cotton growth and physiology. The experiments were conducted in pots, and the waterlogging was initiated by plugging the drain hole at the bottom of the pot using a wooden peg. In the experiments, eight waterlogging treatments, including the control treatment, were imposed at the vegetative growth stage (15 days after sowing). Control treatment had zero days of water-logged condition; other treatments had 2, 4, 6, 8, 10, 12, and 14 days of waterlogging. It took five days to reach zero oxygen levels and one to two days to return to control after the treatment. After a total treatment duration of 14 days (30 days after sowing), the growth, physiological, reproductive, and nutrient analysis was conducted. All physiological parameters decreased with the number of days of waterlogging. Flavonoid and anthocyanin index increased with increased duration of waterlogging. Photosynthesis and whole plant dry weight in continuously waterlogged conditions were 75% and 78% less compared to 0, and 2-day water-logged plants. Plant height, stem diameter, number of main stem leaves, leaf area, and leaf length also decreased with waterlogging duration. When waterlogging duration increased, leaf, stem, and root macronutrients decreased, while micronutrients showed mixed trends. Based on the experimental study, functional relationships (linear, quadratic, and exponential decay) and waterlogging stress response indices are developed between growth and development parameters and the duration of waterlogging. This can serve as a base for developing or improving process-based cotton models to simulate the impact of waterlogging.

## 1 Introduction

Waterlogging is a phenomenon in which the water ponds on the soil surface due to high intensity/duration rainfall, irrigation practices, lateral groundwater flows, poor soil structure, rising/preached water table, or combinations of these factors (Najeeb et al., 2015; Liu et al., 2021). A historical precipitation analysis by the Intergovernmental Panel on Climate Change (IPCC) has indicated a significant increase in high precipitation events in many regions of the world (Singh, 2011; Field et al., 2012). It is estimated that 12% of the world’s arable land could be waterlogged frequently, leading to a reduction of approximately 20% in crop yield (Setter & Waters, 2003; Liu et al., 2023). Waterlogging is one of the major causes of abiotic stress in plants (Pan et al., 2021; Walne and Reddy, 2021). In the United States, flooding was ranked second among the abiotic stresses contributing to crop production losses based on a 12-year (2000-2011) analysis (Bailey-Serres et al., 2012). In the context of an increase in flooding and prolonged waterlogging conditions in many regions that could impact global food production and food security, studying the impact of waterlogging on crops is essential (Liu et al., 2020b).

A global meta-analysis of 115 studies by Tian et al. (2021) observed that cotton crops had the highest reduction in yield (59.95%) compared to other row crops under waterlogging stress (Tian et al., 2021). Previous studies on cotton crops have shown that waterlogging impaired root growth (Meyer et al., 1987; Zhang et al., 2015), nutrient uptake and transport (Milroy et al., 2009), hormonal signaling (Goswami, 1990; Pandey et al., 2003), canopy development, photosynthesis, and radiation use efficiency (Bange et al., 2004; Milroy et al., 2009; Milroy and Bange, 2013; Najeeb et al., 2015) and cotton yield (Kuai et al., 2015; Najeeb et al., 2015; Wang et al., 2017). In cotton, waterlogging in the early growth stage has been found to impact yield, nutrient uptake, and plant growth more significantly than waterlogging in the later crop growth stages (Bange et al., 2004; Milroy et al., 2009; Snider et al., 2018). Though there have been several experimental research on the impact of waterlogging on cotton, a systematic study on the effects of different durations of waterlogging on crop growth and development is not investigated from the point of view of developing functional relationships for modeling the impact of waterlogging on cotton crop.

Some of the common approaches for modeling waterlogging impacts on crop growth and development are by correlating waterlogging with inhibition of biomass accumulation (e.g., Aquacrop (Steduto et al., 2009)), reduction in light interception (e.g., APSIM (Githui et al., 2022)), alterations in carbohydrate accumulation (e.g., SWAGMAN (Shaw and Meyer, 2015)), delaying phenological processes, etc. (Liu et al., 2020b; Yan et al., 2022). Similarly, for developing/improving a cotton model for waterlogging simulations, obtaining functional relationships between waterlogging and all the critical plant growth and development parameters are essential. Besides determining primary plant growth and development parameters, a mechanistic process-based model would also require additional information on processes influenced by waterlogging (e.g., root water and nutrient uptake, understanding the effect of waterlogging on plants’ micro and macronutrient status, soil oxygen concentration, plant pigments, and stress response components, etc.) under varying waterlogged conditions. Currently, most waterlogging studies are limited to analyzing plant growth and development under control conditions (no-waterlogging stress) versus fully waterlogged conditions (An et al., 2016; Ikram et al., 2022; Teoh et al., 2022). Experiments with different duration of waterlogging are necessary to generate dynamic temporal relationships between waterlogging and various plant-related features (Wang et al., 2017). Some of the water-logging studies are based on field experiments, which are location specific and affected by confounding factors (e.g., nutrient availability) and poor field trial reproducibility (Bange et al., 2004; Liu et al., 2020a; Milroy et al., 2009; Walne & Reddy, 2021). Therefore, a comprehensive controlled experimental investigation should be carried out under controlled environmental and management conditions for varying waterlogging durations to develop standard functional relationships.

The present study aims to establish functional relationships between waterlogging and cotton growth and development-related features. As an initial step towards developing functional relationships and since early crop growth is significantly affected by waterlogging stress compared to later growth stages (Bange et al., 2004; Milroy et al., 2009), the current study is focused on analyzing early-stage cotton growth and development. Specific objectives of the study are to (a) quantify the impacts of different durations of waterlogging on pigments, root morphology, shoot morphology, reproductive performance, and macro and micronutrients and (b) develop functional relationships and water stress response indices between the duration of waterlogging, and growth and development parameters. This study hypothesizes that waterlogging stress during the early stages of cotton growth significantly impacts various growth and development-related features. It is anticipated that longer waterlogging durations will have a more pronounced negative effect on the measured variables.

## 2 Materials and methods

### 2.1 Experimental facilities and setup

The experiments were carried out at the Environmental plant physiology laboratory at the Mississippi Agricultural and Forestry Experimentation Station, Mississippi State University, MS, USA (33° 28’ N, 88° 47’ W). The plants were grown in polyvinylchloride pots (diameter 15.2 cm and 30.5 cm long; 5.5 L volume). A drain hole was made on the bottom side of the pots to facilitate water drainage. The bottom one inch of the pot was filled with 500 g of clean pea gravel, and the rest was filled with a 3:1 ratio of fine sand (particle size less than 0.3 mm) and ground topsoil (87% sand, 11% silt, and 2% clay). Irrigation and nutrients were supplied such that the pots were maintained at field capacity, along with nutrients made available non-limiting for plant uptake. Throughout the experiments, pots were irrigated thrice per day with full-strength Hoagland’s nutrient solution for 90 seconds (Hewitt, 1952). Rain shelters (mini hoop houses) with clear plastic were placed over the pots to prevent the impact of precipitation. The cotton cultivar DP 1646 B2XF was used in this study. This is a mid-full season cultivar with medium tall, smooth leaf type, accounting for 20.3% of total US cotton acreage. Four cotton seeds were sown in each pot and thinned to one plant per pot upon emergence. The average ambient air temperature during the experimental period was 33.3 ± 2.3 ˚C. The maximum, minimum, and average daily solar radiation observed during the duration of the experiments were 27.5, 10.4, and 21.7 MJ m^-2^ d^-1^, respectively, with an average day length of 14 hours (Delta agricultural weather center, MSU).

### 2.2 Waterlogging treatments

Waterlogging treatments were imposed once two true leaves appeared above the cotyledons. This was around 15 days after sowing. Waterlogging was initiated by plugging the drain hole in the pot using a wooden peg (Pan et al., 2021; Walne and Reddy, 2021). The experiments were carried out with eight waterlogging treatments, including a control. Water logging was not imposed in the control treatment. The other treatments’ designated days of flooding (waterlogging) were 2, 4, 6, 8, 10, 12, and 14 days. Five experimental replications were made for each of the treatments. Five cm of water was maintained during waterlogging. The water levels were monitored twice daily to ensure waterlogging was continuously imposed over the designated flooding period. Oxygen content was measured using Apogee SO-110 soil oxygen sensors (Apogee Instruments, Inc, Logan, UT, USA). The oxygen sensors were inserted 5 to 8 cm from the soil surface. The wooden plug in the drain hole was released to stop the waterlogging treatments. The pots were maintained at field capacity for the rest of the duration of the experiment.

### 2.3 Measurements

The following plant growth and development parameters were measured at the end of the waterlogging treatment (14 days after the treatment or 30 days after sowing)

#### 2.3.1 Plant physiology

Photosynthesis, stomatal conductance, intercellular carbon dioxide (CO_2_) concentration, and fluorescence were measured in all plants using the LI-6800 photosynthesis system. The temperature was set at 30°C, CO_2_ at 420 ppm, PAR at 1500 µmol m^-2^ s^-1^, and relative humidity at 60% while taking the measurements. The measurements were taken on the 3^rd^ or 4^th^ leaf from the top of each plant. PSII effective chlorophyll fluorescence (Fv'/Fm') was calculated using the equation; (Fv'/Fm')=(Fm'-Fo')/Fm', where Fm' is the maximal fluorescence of light-adapted leaves, Fo' is the minimal fluorescence of a light-adapted leaf that has momentarily been darkened for 30 min using the Li-Cor dark adapting clips (Genty et al., 1989; Kakani et al., 2008). The electron transport rate (ETR) was calculated using the equation ((Fm' - Fs)/Fm') * fIα_leaf_, where Fs is the steady state fluorescence, and f is the fraction of absorbed quanta that Photosystem II uses, I is the incident photon flux density (µmole m^-2^ s^-1^), and α_leaf_ is the leaf absorptance. PSII actual photochemical quantum yield or photosystem efficiency (PhiPS2) was calculated using the equation (Fv'-Fs)/Fm', where Fs is the steady state fluorescence. Nonphotochemical chlorophyll fluorescence quenching (NPQ) is estimated using the equation (Fm' - Fs)/(Fm' - Fo') (Rosenqvist and van Kooten, 2003; Surabhi et al., 2009)

#### 2.3.2 Pigment estimation

Leaf chlorophyll content, flavonoid index, anthocyanin, and nitrogen balance index were measured using a hand-held Dualex^®^ scientific instrument (Force A DX16641, Paris, France). These were measured for the recently fully expanded uppermost leaves on the plant.

#### 2.3.3 Root and shoot morphology

The number of mainstem leaves was measured as the total number of fully developed leaves. Leaf area was measured using the LI-3100 leaf area meter (Li-COR, Inc., Lincoln, NE). The stem diameter and leaf length were measured using a digital caliper and standard matric ruler respectively. The plant’s above-ground parts were removed, and the root system and soil media were carefully collected from each pot. This was washed using a gentle stream of water until the roots were clean. The roots were then carefully untangled using plastic forceps after placing them in a 400 cm long and 300 cm wide acrylic tray with 5 mm of water. This process helped in minimizing root overlap. Root images were captured using Epson Expression 11000XL scanner at 800 dpi resolution (Epson America, Inc., Long Beach, CA, USA). Images were analyzed by WinRHIZO Pro 2009C software (Regent Instruments, Inc., Québec, QC, Canada) to extract the number of root tips, root crossings, total root length, root surface area, and root volume.

All plant parts were separated and collected in separate bags. The samples were oven dried at 80˚C to ensure a constant weight. The dry weights of leaf, stem, and root were to summed to get the whole plant’s dry weight (g plant^-1^). Plant height was measured using a standard matric ruler as the distance between the cotyledonary node to the topmost unfolded mainstem leaf.

#### 2.3.4 Reproductive growth

The number of days from emergence to the first square was recorded for plants in all the waterlogging treatments.

#### 2.3.5 Macro and micronutrients

Macronutrients (nitrogen, calcium, potassium, magnesium, phosphorous, sulfur) and micronutrients (boron, copper, iron, manganese, and zinc) in the leaf, stem, and root of the cotton plant was measured 14 days after the treatment (30 days after sowing). Using standard protocols, these were measured at the Soil Testing Laboratory at Mississippi State University, Mississippi State, MS, USA (Plank, 1992).

#### 2.3.6 Waterlogging stress response index

Walne and Reddy (2021) developed ‘water stress response index (WSRI)’ based on the concept of the environment productivity index initially introduced by Nobel (1984) (Nobel, 1984; Reddy et al., 2008; Walne and Reddy, 2021). This was used for a normalized representation of the impact of flood stress on each of the measured plant growth and development-related features on a comparable scale (Walne and Reddy, 2021). The WSRI is estimated by normalizing each parameter’s mean-observed values by dividing the mean values under stress by the value of the respective parameter under the 0 days treatment (no stress or control conditions). The WRSI values range from 0 (where waterlogging is fully limiting) to 1 (where waterlogging stress is not limiting the parameter). The index values were derived using the equation,where is the waterlogging stress response index of parameter attreatment, is the value of parameter at 0 days of waterlogging, andis the parameter at treatment.

### 2.4 Statistical analysis

A SAS program PROC GLM was used to analyze variance among the effects of all eight waterlogging treatments on all the measured cotton crop growth and development parameters. Graphs and functional relationships were developed using SigmaPlot 13 (Systat Software, Inc, San Jose, CA, USA) and RStudio programming (version 2022.12.0).

## 3 Results and discussions

**Figure 1:**
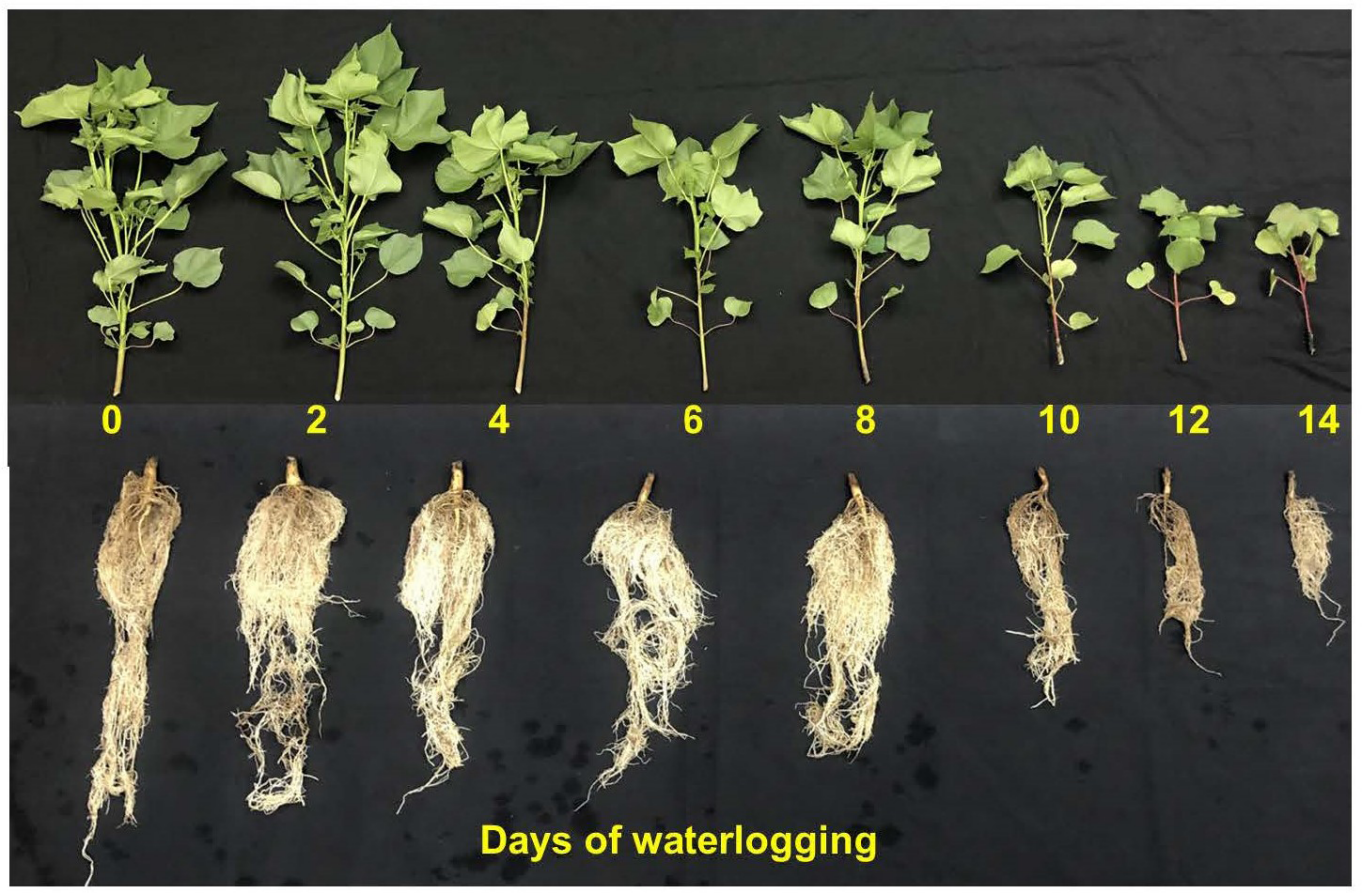
Pictorial representation of cotton shoot and root growth and development measured 14 days after treatment. The numbers in the figure represent the days the plant was subjected to waterlogging treatments.

The 14 days of waterlogged cotton plants showed smaller and thinner leaves with scorching symptoms compared to the control. The root, in terms of its dry weight, length, surface area, root tips, and crossings, decreased with an increase in the number of days of waterlogging. In most plants under waterlogged conditions, root growth is affected since the anoxic/hypoxic condition can result in lower root respiration and root vigor, death of root cells, reduction in root permeability, etc. (Christianson et al., 2010; Liu et al., 2015; Najeeb et al., 2015; Liu et al., 2021). There were gradients in stem color and petioles from control to water-logged plants (**Figure 1**). The intensity of the dark brown pigmentation increased with days of waterlogging, possibly due to stress-protective components produced in plants under stress (e.g., anthocyanins and flavonoids, etc.) (Long et al., 2019). Continuous waterlogging has also resulted in the formation of hypertrophic lenticels at the base of the roots that have lodged the plants. The formation of hypertrophic lenticels that provide a greater surface area around submerged portions of the stem base facilitates quick gas exchange and has been observed in oxygen-stressed roots of several plants (Hook, 1978; Jackson & Ricard, 2003; Liu et al., 2020b). Overall, water logging has significantly affected all the plant growth and development-related features (**Table S1, supplementary file**). Each measured growth and development parameter in this study is discussed in detail in the following sections.

### 3.1 Soil oxygen levels

**Figure 2:**
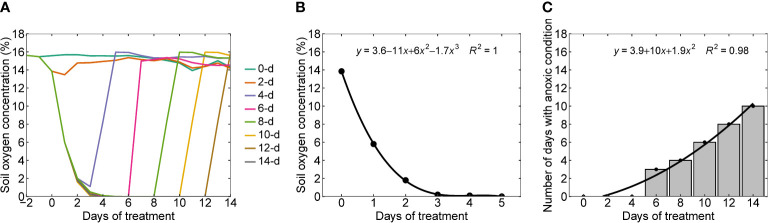
**(A)** Temporal trends in soil oxygen levels in all eight waterlogging treatments (0 (control), 2, 4, 6, 8, 10, 12, and 14 days of waterlogging), **(B)** oxygen response curve or functional relationship between the soil oxygen concentration and duration of waterlogging, and **(C)** the number of days with an anoxic condition in all the treatments. Waterlogging treatments were imposed once two true leaves appeared above the cotyledons.

The soil oxygen levels decreased with an increase in the number of days of waterlogging (**Figure 2A**). Except for two and four-day waterlogging treatments, all other treatments have reached zero oxygen concentration levels in the soil or anoxic conditions. On average, it took five days to reach zero oxygen levels for the rest of the treatments. Once the waterlogging treatment was stopped, the soil oxygen level returned to the control level within one to two days. **Figure 2B** presents the functional relationship (oxygen response curve) between the duration of waterlogging (x) and soil oxygen concentration (y) specific to the soil considered in the experiments. **Figure 2C** presents the number of days with anoxic conditions in all eight waterlogging treatments and their association with days of treatment, which followed a quadratic functional relationship.

Some of the general approaches to define the threshold for waterlogging stress are; when the water table is within 30 cm from the soil surface (Bakker et al., 2007), when the soil oxygen concentration becomes less than 10% air-filled pore space (Kramer and Boyer, 1995), or when the available water fraction in the soil surface layer is at least 20% higher than the field water capacity (Aggarwal et al., 2006). Some of the waterlogging stress quantification approaches are the excess water table duration approach (Sieben, 1964; Wesseling, 1974), surface and subsurface water excess (Shen et al., 1999), stress day index (Hiler et al., 1974), which measure waterlogging stress according to the depth of the water table and duration of the waterlogging period. A more reasonable approach for correlating the impact of waterlogging on the crop is the oxygen response curve in the root zone because it’s the reduction in the oxygen concentration in the soil that significantly affects the soil chemical properties, soil enzymes, phytotoxicities, alteration to root growth, root water and nutrient uptake (Liu et al., 2020b; Shaw & Meyer, 2015). In addition, variation in soil oxygen concentration is more representative of soil features as soil oxygen dynamics is influenced by soil texture and structure (Neira et al., 2015).

### 3.2 Nutrient analysis

**Figure 3:**
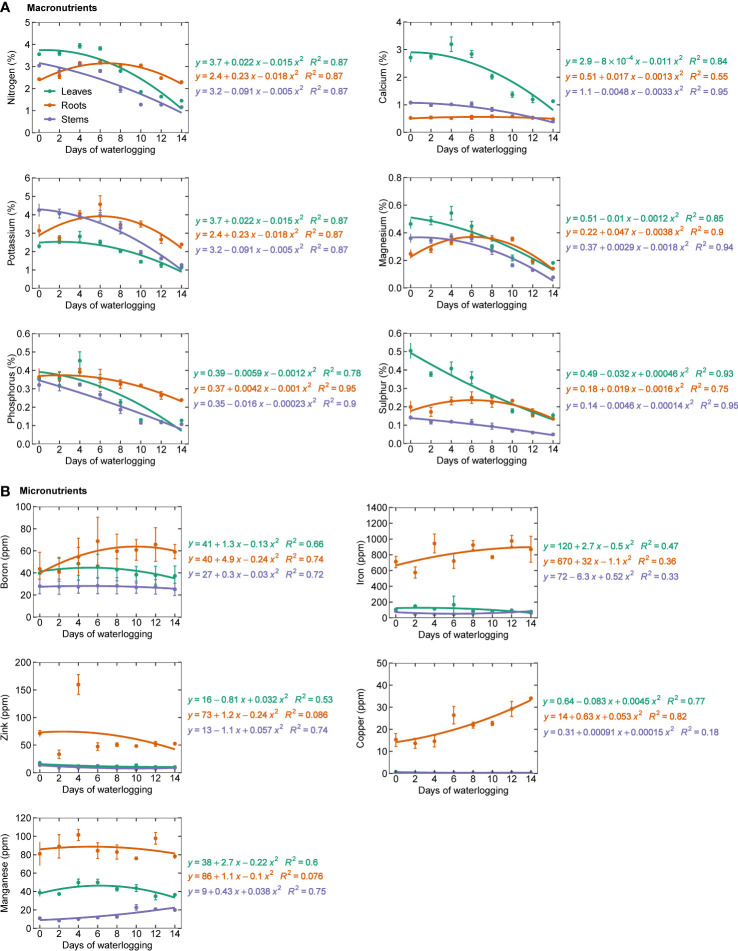
Macronutrients **(A)** (nitrogen, calcium, potassium, magnesium, phosphorous, and sulfur) and **(B)** micronutrients (boron, copper, iron, manganese, and zinc) in leaf, stem, and roots.

All the measured macronutrients were observed to be decreasing with an increase in the number of days of waterlogging **Figure 3A**. Similar observations were made by (Hocking et al., 1985; Hocking et al., 1987) and Milroy et al. (2009) (Hocking et al., 1985; Hocking et al., 1987; Milroy et al., 2009). Waterlogging induces a hypoxic/anoxic condition in the soil and reduces root respiration and oxygen concentration in the root tissues. Once oxygen concentrations in root tissues drop below the critical oxygen pressure for respiration, they become oxygen-limited (Armstrong and Drew, 2002). This can result in the death of root cells or a decrease in root permeability, or complete death of roots, which can lead to reduced nutrient uptake. Reduction in the permeability of the roots to water and nutrients results in stomatal closure, which can further enhance the decline in nutrient uptake (Sojka and Stolzy, 1980). Hodgson and MacLeod (1988) observed a reduction in nitrogen uptake despite the heavy application of nitrogen fertilizer in soil (Hodgson and MacLeod, 1988). Waterlogging can also alter the cation exchange capacity of soil particles and the valency of nutrients, making them toxic or unavailable for plant uptake (Setter et al., 2009; Bailey-Serres and Colmer, 2014). Waterlogging can also result in low energy in the roots, which prevents the movement of nutrients to the other plant organs (Ashraf et al., 2011). Since an overall decrease is observed in the macronutrients in the present study, the impact of waterlogging on macronutrients could be due to macronutrient deficiency rather than nutrient toxicity (Steffens et al., 2005). Micronutrients showed a mixed trend (increase/decrease) in the leaf, stem, and roots. Copper, manganese, and iron were found to increase in stem with an increase in waterlogging duration (**Figure 3B**). Except for zinc and manganese, all other micronutrients showed a decreasing trend in roots. In leaves, all the micronutrients were found to decrease with an increase in waterlogging duration. Zhang et al. (2021b) concluded that waterlogging inhibits the uptake of most of the major nutrients but also increases some of the essential nutrients. Therefore, in addition to the decrease in plant nutrients, the nutrient imbalance in the plant could also eventually lead to reduced plant growth and yield (Zhang et al., 2021b).

### 3.3 Gas exchange, photochemical, and fluorescence parameters

**Figure 4:**
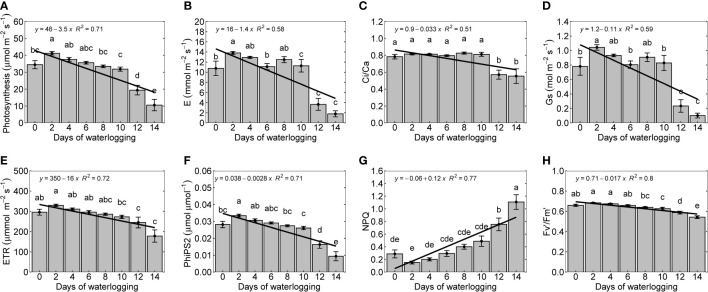
Leaf photosynthesis **(A)**, transpiration, E **(B)**, the ratio of intercellular and ambient CO_2_ concentrations (C_i_/C_a_) **(C)**, stomatal conductance (G_s_) **(D)**, Photosynthetic electron transport rate (ETR) **(E)**, PSII actual photochemical quantum yield or photosystem efficiency (PhiPS2) **(F)**, nonphotochemical chlorophyll fluorescence quenching (NPQ) **(G)**, and PSII effective chlorophyll fluorescence (Fv’/Fm’) **(H)** measured 14 days after treatment. The lower-case letters denote statistically significant differences (P < 0.01) between treatments according to Fisher’s LSD test.

Photosynthesis was observed to decline with an increase in the duration of waterlogging. Continuously waterlogged plants showed 75% less photosynthesis than 0 and 2-day water-logged plants (**Figure 4A**). The photosynthesis decline can be related to a decrease in transpiration (E) and stomatal conductance (G_s_), which reduces the supply of CO_2_ to the intercellular space (**Figures 4B–D**). Under waterlogged conditions, the permeability of the root to water uptake decreases, resulting in stomatal closure in cotton (Sojka and Stolzy, 1980). It can also be associated with non-stomatal limitations, as the ratio of intercellular and ambient CO_2_ concentrations (Ci/Ca) declined under 12 and 14-day waterlogged conditions (**Figure 4C**). The decline in photosynthesis could also be due to a decrease in leaf nitrogen concentration due to the reduced nutrient uptake in water-logged conditions (Jackson, 1984) (**Figure 3A**). The reduction in photosynthesis and carbon assimilation alters the formation of new leaves, further enhancing the reduction in photosynthesis. The results from this study are consistent with other studies that reported a decrease in photosynthesis, transpiration, and stomatal conductance with an increase in waterlogging (Milroy and Bange, 2013; Najeeb et al., 2016; Zhang et al., 2016). Previous studies have correlated the reduction of photosynthesis with a decrease in the stability of leaf thylakoids (Luo et al., 2008), a decrease in the stomatal conductance (Yordanova et al., 2005; Christianson et al., 2010; Najeeb et al., 2015), reduction in leaf chlorophyll content, transpiration rate, and leaf water potential (Meyer et al., 1987), and reduced activity of Rubisco (Pandey et al., 2000).

PSII actual photochemical quantum yield or photosystem efficiency (PhiPS2) and PSII effective chlorophyll fluorescence (Fv'/Fm') decreased with an increase in the duration of waterlogging (**Figures 4F, H**). Photosynthetic electron transport rate (ETR) that represents the photosynthesis efficiency and the ability to produce adenosine triphosphate (ATP) and NADPH (nicotinamide adenine dinucleotide phosphate) from light energy decreased with an increase in the duration of waterlogging (**Figure 4E**). This is due to the reduction in the PhiPS2 and Fv'/Fm' which affect electron transport and changes the amount of light directed into organic synthesis (Zhang et al., 2019). Early reduction in photosynthesis of waterlogged plants could be due to internal damage to photosystem II associated with photoinhibition and is independent of stomatal closure (Ahmed et al., 2006). Nonphotochemical chlorophyll fluorescence quenching (NPQ) increased with waterlogging duration (**Figure 4G**). NPQ represents the thermal energy not used in photochemistry and photochemical damage caused by stresses (Maxwell and Johnson, 2000). Waterlogging can lead to oxygen deprivation in the roots and tissues of plants. Reduced oxygen availability can inhibit the production of ATP and NADPH, which are required for photosynthesis. This results in excess energy being accumulated in chlorophyll molecules, forcing plants to engage in NPQ to dissipate the energy.

### 3.4 Pigment estimation

**Figure 5:**
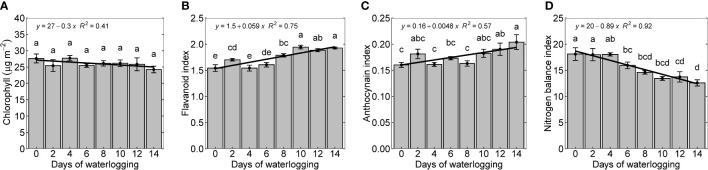
Chlorophyll **(A)**, flavonoid index **(B)**, anthocyanin **(C)**, and nitrogen balance index **(D)** measured 14 days after treatment. The lower-case letters denote statistically significant differences (P < 0.01) between treatments according to Fisher’s LSD test.

Leaf chlorophyll represents the quality and productivity of the crop and is generally used as an index to diagnose diseases, plant nutrients, and nitrogen status (Dey et al., 2016). Though there were no considerable differences in chlorophyll measured after 14 days, chlorophyll content in all treatments was slightly lower (12.2% lower) than in the control treatment (**Figure 5A**). This is similar to previous studies, which observed decreased chlorophyll content with increased waterlogging (Pan et al., 2021; Zhang et al., 2021a). Ren et al. (2016) observed a reduced chlorophyll content in the plants due to changes in the chloroplast structure and morphology with waterlogging (Niki et al., 1978; Ren et al., 2016; Zhang et al., 2019).

The flavonoid and anthocyanin index represents stress-protective components that are produced in plants during stress (abiotic stress, pathogen invasion, antioxidation, etc.) (Panche et al., 2016; Long et al., 2019; Qin et al., 2021). Flavonoid and anthocyanin index increased linearly with increasing duration of waterlogging, indicating that its production increased as part of plants’ protective mechanisms (**Figures 5B, C**). The percentage increase in flavonoid and anthocyanin between control and 14 days of waterlogging was 24.8 and 28.7%, respectively. The increase in the intensity of the dark brown pigmentation with an increase in days of waterlogging indicates the elevated presence of anthocyanins (**Figure 1**). The increased flavonoid and anthocyanin index agrees with previous studies that showed a rise in stress-protective components in response to various plant stresses (Chalker-Scott, 1999; Merzlyak and Chivkunova, 2000; Winkel-Shirley, 2002; Walne and Reddy, 2021).

The nitrogen balance index (NBI) is the ratio of chlorophyll to epidermal flavonoids. NBI is less sensitive to phenology and reflects nitrogen availability better than either chlorophyll content or epidermal polyphenols (Cerovic et al., 2012; Fan et al., 2022). With increased waterlogging duration, NBI declined linearly, indicating decreased nitrogen uptake (**Figure 5D**). As observed in this study, the decrease in nitrogen content in the plant due to waterlogging supports these findings (**Figure 3A**). Even though the experiment was conducted at a non-limiting nutrient condition, a reduction in N uptake shows the impact of the waterlogged system on N uptake. A similar observation was made by Hodgson & MacLeod (1988) (Hodgson & MacLeod, 1988).

### 3.5 Root and shoot morphology

**Figure 6:**
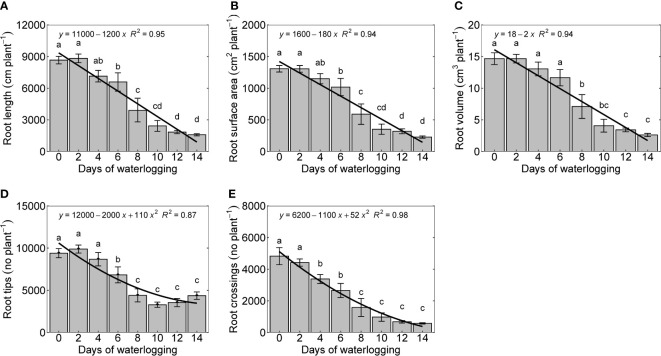
Root length **(A)**, root surface area **(B)**, root volume **(C)**, number of root tips **(D)**, and number of root crossings per plant **(E)** measured 14 days after treatment. The lower-case letters denote statistically significant differences (P < 0.01) between treatments according to Fisher’s LSD test.

Roots control water and nutrient uptake and transport to the above-ground organs and play a significant role in the synthesis of hormones regulating plant response to waterlogging (Najeeb et al., 2015). In this study, measured root growth parameters, root length, root surface area, and root volume declined linearly with an increase in the number of days of waterlogging. The percentage decrease in root length, surface, and area root volume were 70, 86, and 95, respectively (**Figures 6A–C**). Root tips and root crossings were quadratically reduced with the number of days of waterlogging (**Figures 6D, E**). Waterlogging changes the energy metabolism of cotton plants from aerobic to anaerobic respiration, affecting root growth (Zhang et al., 2021a). Cotton roots are intolerant of low oxygen concentrations, and it inhibits root growth even in a mildly hypoxic condition (oxygen concentration <10%) (Meyer et al., 1987). Huck (1970) observed a complete death of terminal apices of the cotton roots with just three hours of anoxic conditions (Huck, 1970). An overall reduction in the root growth and its functioning is caused by a complex set of processes in the root (inhibition of cell division, transitory cessation of tap roots elongation, death of terminal apices, damage of root tissues, reduction in the ATP synthesis), which primarily starts from the root respiratory impairment (Najeeb et al., 2015).

**Figure 7:**
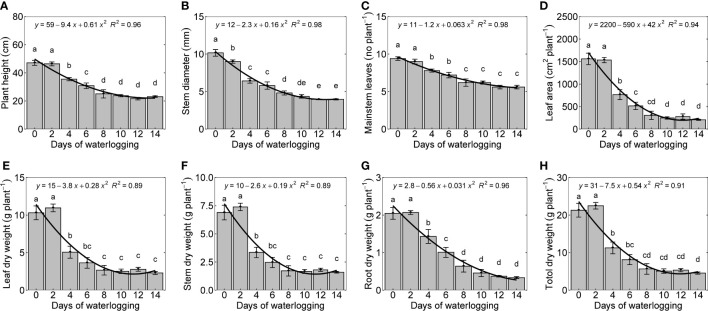
Waterlogging effect on cotton shoot growth and development [plant height **(A)**, stem diameter **(B)**, number of mainstem leaves **(C)**, leaf area **(D)**, dry weight of leaf **(E)**, stem **(F)**, root **(G)**, whole plant weight **(H)**] measured 14 days after treatment. The lower-case letters denote statistically significant differences (P<0.01) between treatments according to Fisher’s LSD test.

Plant height, stem diameter, mainstem leaves, and leaf area declined quadratically with an increase in the number of days of waterlogging (**Figures 7A–D**). The number of mainstem leaves in the control experiments was found to be 9.4, which is similar to the number of mainstem leaves reported in a previous experimental study by Reddy et al. (1992) conducted under a similar temperature range as the present study (Reddy et al., 1992). Plant height, stem diameter, mainstem leaves, and leaf area were decreased by 55%, 61%, 40.4%, and 88%. Leaf, stem, root dry weight, and whole plant weight decreased with an increase in days of waterlogging (**Figures 7E–H**). The percentage decrease in leaf, stem, and root dry weight was 77, 76, and 83, respectively. Reduced plant growth is a typical response to waterlogging (Bange et al., 2004; Milroy and Bange, 2013; Zhang et al., 2015; Zhang et al., 2016). A similar decrease in cotton root and shoot growth has been observed in previous studies on waterlogging (Bange et al., 2004; Milroy et al., 2009; Najeeb et al., 2015).

**Figure 8:**
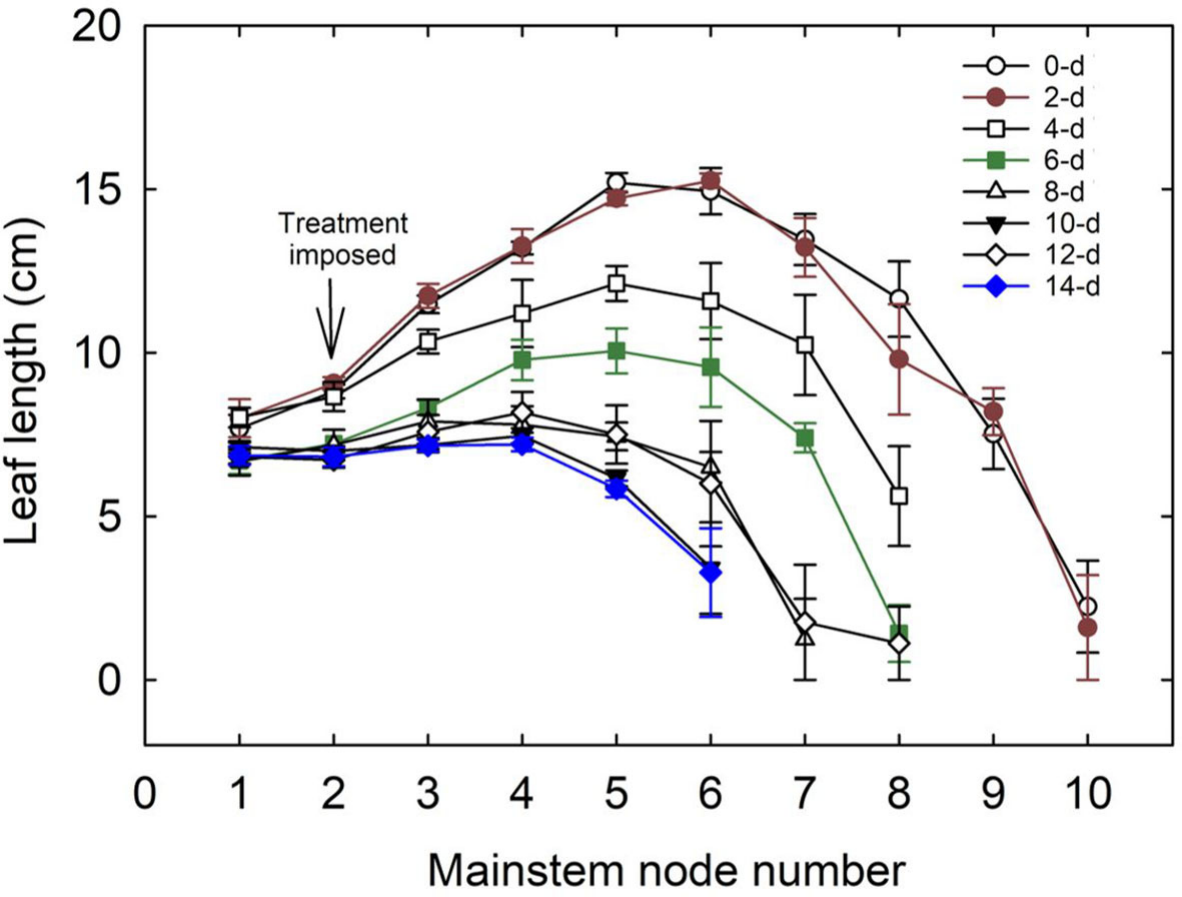
Variation in the length of leaves on the main stem nodes with the number of days of waterlogging. 0-d, 2-d,4-d, 6-d, 8-d, 10-d, 12-d, and 14-d represent the number of days of water logging. The downward arrow indicates the initiation of the waterlogging treatment, which was started when the plants had two true leaves above the cotyledons.

In cotton, the leaves on mainstem nodes feed the developing terminal, branches, and bolls and are significantly involved in the structural development of the plant (Oosterhuis and Urwiler, 1988). The number of mainstem nodes decreased with an increase in the duration of waterlogging. Plants in control and two-day treatment produced ten mainstem nodes and leaves. The 12 and 14-day waterlogging treatments produced only six mainstem nodes per plant. A reduction in leaf length was observed with an increase in the days of waterlogging (**Figure 8**). The control (0-day) treatment produced a maximum leaf length of 15 cm, whereas 12 and 14-day water-logged conditions had a maximum leaf length of only 7 cm. The difference in the leaf lengths between the leaves on the corresponding mainstem nodes in control and waterlogged scenarios increased with an increase in node number. In 14 days of waterlogging treatment, leaves were produced only up to node number 6. Leaf size and canopy architecture are major determinants of the absorption of incoming photosynthetic active radiation (Najeeb et al., 2015). This shows the extent of the influence of waterlogging on leaf growth, which will result in a cascading effect on transpiration, photosynthesis, and overall plant growth.

### 3.6 Reproductive growth

**Figure 9:**
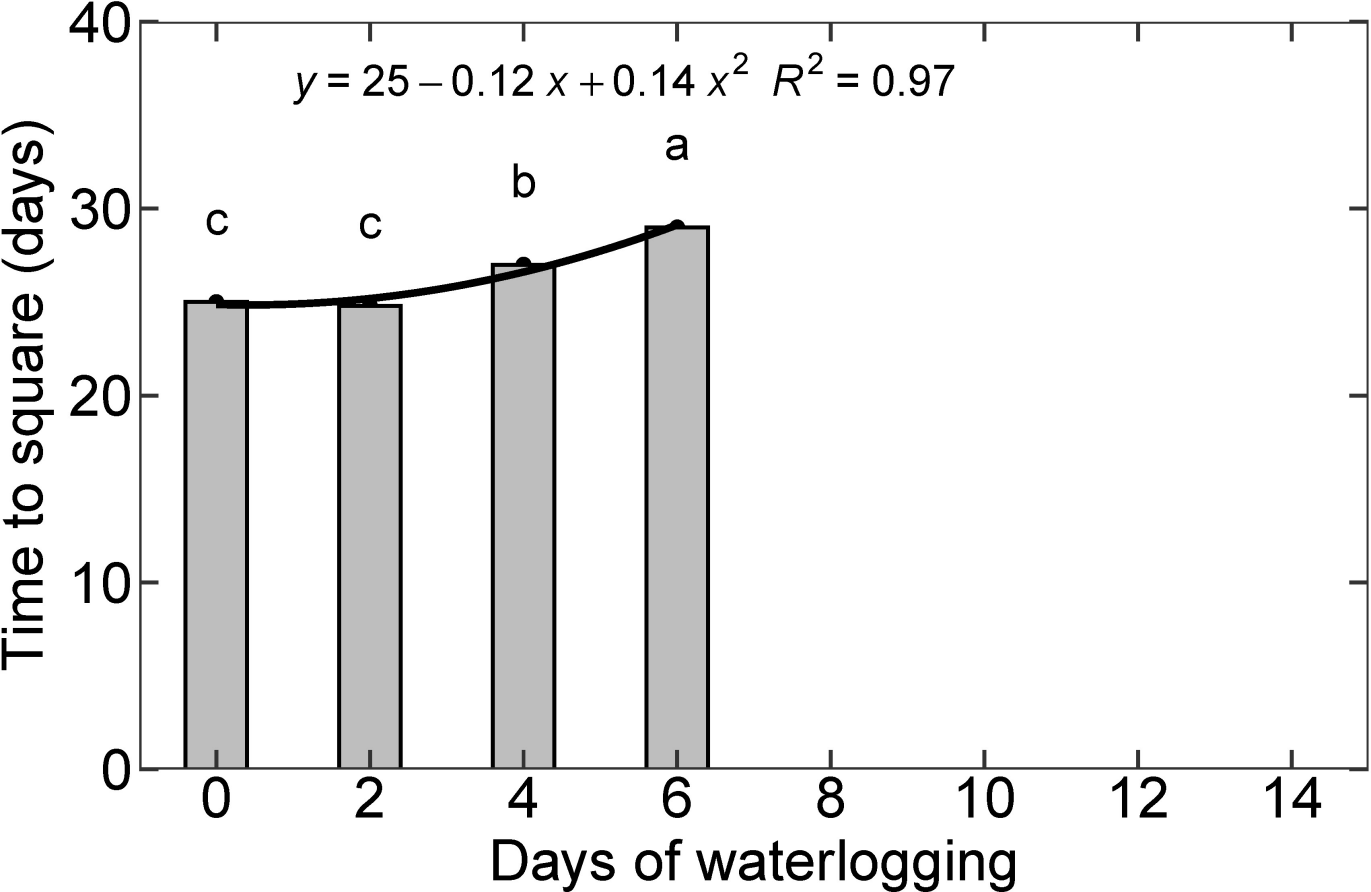
Time to square for all eight treatments. The lower-case letters denote statistically significant differences (P < 0.01) between treatments according to Fisher’s LSD test.

The first square is usually visible about 25-38 days after planting on nodes 5 to 7 (Oosterhuis, 1990). In the control (with no waterlogging treatment) and two-day waterlogging treatment, it took 25 days to square similar to the results presented by Reddy et al. (1992); under optimum water, nutrient, and temperature conditions. Four and six days of waterlogging delayed square formation by two and four days, respectively. The other treatments (8, 10, 12, and 14 days of waterlogging) did not produce squares during the 30-day experimental period. Based on the functional relationship between the days of waterlogging and time to the first square (**Figure 9**), the 8, 10, 12, and 14 treatments would develop the first square on 32, 37, 43, and 50 days, respectively. A delay in the first square formation can reduce the number of bolls formed on the plant, resulting in a lower cotton yield. Zhang et al. (2016) observed a yield reduction of 57%, 27.2%, and 12.1% during waterlogging at the squaring, flowering, and boll settings, respectively (Zhang et al., 2016). Several studies have related the reduction in cotton yield under waterlogging to suppressed dry matter production that leads to a decrease in boll numbers (Hodgson and Chan, 1982; Bange et al., 2004; Kuai et al., 2015; Zhang et al., 2016). In most studies, a higher yield reduction was observed when waterlogging was imposed at the early growth stage compared to the flowering or boll setting stage (Bange et al., 2004; Zhang et al., 2016).

### 3.7 Waterlogging stress response index

**Figure 10:**
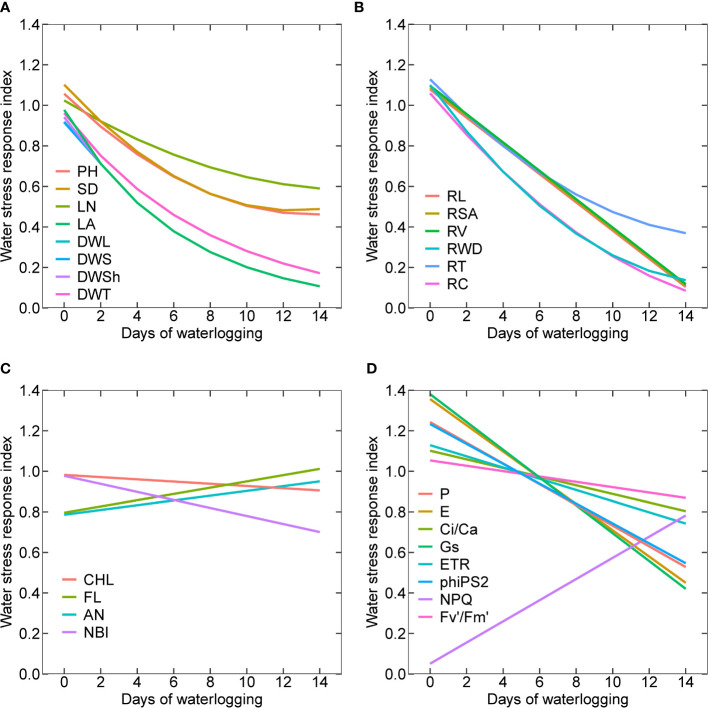
Waterlogging stress response indices for all the shoot **(A)** and root morphological parameters **(B)**, plant pigments **(C)**, gas exchange, photochemical and fluorescence parameters **(D)**; plant height (PH), stem diameter (SD), mainstem leaf number (LN), leaf area (LA), dry weight of leaves (DWL), dry weight of stems (DWS), dry weight of shoot (DWSh), dry weight of whole plant (DWT), root length (RL), root surface area (RSA), toot volume (RV), toot dry weight (RWD), toot tips (RT), and toot crossings (RC), chlorophyll (CHL), flavonoid index (FL), anthocyanain index (AN), nitrogen balance index (NBI), photosynthesis (P), transpiration (E) ratio of intercellular and ambient CO_2_ concentrations (Ci/Ca), stomatal conductance (Gs), photosynthetic electron transport rate (ETR), PSII actual photochemical quantum yield or photosystem efficiency (PhiPS2), nonphotochemical chlorophyll fluorescence quenching (NPQ) and PSII effective chlorophyll fluorescence (Fv’/Fm’).

Quantifying the relative response of parameters to waterlogging can help understand the impact of stress on plant performance. Waterlogging stress response index (WSRI) is determined for all the shoot (**Figure 10A**) and root morphological parameters (**Figure 10B**), plant pigments (**Figure 10C**), and gas exchange, photochemical, and fluorescence parameters (**Figure 10D**). The regression equations and parameters are presented in **Table 1**. The experiments with zero days of waterlogging represent cotton’s potential growth and development. Among all the shoot morphological parameters, leaf number had the lowest relative decline, followed by plant height, stem diameter, dry weight of different plant parts, and leaf area (**Figure 10A**). This shows that the leaf area was affected more than the number of leaves. The decline in waterlogging stress response indices in plant height and stem diameter was found to be similar. For dry weight and leaf area, a higher reduction in the WSRI was observed at a lower number of days of waterlogging (0 to 6 days) compared to more than six days (6 to 14 days). The average decline in WSRI from 0 to 6 days and from 6 to 14 days was 0.083 WRSI/day and 0.0375 WSRI/day, respectively. Except for root dry weight, root crossings, and the number of root tips, all other root morphological parameters linearly declined with increased waterlogging. The minimum and maximum declines were observed for root tips and crossings.

**Table 1:** Regression parameters and coefficients of waterlogging stress response indices for all the shoot and root morphological parameters, plant pigments, and gas exchange, photochemical and fluorescence as a function of waterlogging duration.

Parameter	Description	*a*	*b*	*c*	R^2^	Equation/function
**PH**	Plant height	0.0032	-0.087	1.056	0.96	Quadratic equation
**SD**	Stem diameter	0.0039	-0.098	1.101	0.98	Quadratic equation
**LN**	Mainstem leaf number	0.0017	-0.054	1.023	0.97	Quadratic equation
**LA**	Leaf area	0.9770	-0.158	–	0.92	Exponential decay function
**DWL**	Dry weight of leaves	0.9188	-0.118	–	0.85	Exponential decay function
**DWS**	Dry weight of stems	0.9157	-0.118	–	0.84	Exponential decay function
**DWSh**	Dry weight of shoot	0.9410	-0.121	–	0.87	Exponential decay function
**DWT**	Dry weight of whole plant	0.9611	-0.123	–	0.88	Exponential decay function
**RL**	Root length	-0.0696	1.078	–	0.94	Linear equation
**RSA**	Root surface area	-0.0698	1.087	–	0.93	Linear equation
**RV**	Root volume	-0.0699	1.096	–	0.94	Linear equation
**RWD**	Root dry weight	0.0038	-0.122	1.1	0.95	Quadratic equation
**RT**	Root tips	0.0028	-0.093	1.128	0.86	Quadratic equation
**RC**	Root crossings	0.002	-0.107	1.059	0.98	Quadratic equation
**CHL**	Chlorophyll	0.98	0.0055	–	0.75	Linear equation
**FL**	Flavonoid index	0.80	0.015	–	0.41	Linear equation
**AN**	Anthocyanin	0.79	0.012	–	0.57	Linear equation
**NBI**	Nitrogen balance index	0.98	0.020	–	0.74	Linear equation
**P**	Leaf photosynthesis	1.2	-0.051	–	0.59	Linear equation
**E**	Transpiration	1.4	-0.065	–	0.58	Linear equation
**Ci/Ca**	Ratio of intercellular and ambient CO_2_ concentrations	1.1	-0.021	–	0.51	Linear equation
**Gs**	Stomatal conductance	1.4	-0.069	–	0.59	Linear equation
**ETR**	Photosynthetic electron transport rate	1.1	-0.028	–	0.72	Linear equation
**phiPS2**	PSII actual photochemical quantum yield or photosystem efficiency	1.2	-0.049	–	0.71	Linear equation
**NPQ**	Nonphotochemical chlorophyll fluorescence quenching	0.051	0.052	–	0.77	Linear equation
**Fv'/Fm'**	PSII effective chlorophyll fluorescence	1.1	-0.013	–	0.80	Linear equation

Linear equation, quadratic equation, and exponential decay function are represented as *y* = *ax* +*b*, *y* = *ax^2^* +*bx+c*, and *y* = *a e^bx^*, respectively, where *y* is the plant parameter, and *x* is the duration of waterlogging in days.

The WSRI of the flavonoid index and anthocyanin index increased with an increase in waterlogging duration. A higher increase was observed for flavonoids than anthocyanins. The WSRI for chlorophyll and NBI decreased with waterlogging duration, with a higher decrease for NBI than chlorophyll (**Figure 10C**). Among all the gas exchange, photochemical and fluorescence parameters, except for the NPQ, the WSRI of all other parameters decreased with an increase in the waterlogging duration. The maximum decline in the WSRI was for stomatal conductance (Gs), and the minimum decrease was for PSII effective chlorophyll fluorescence (Fv'/Fm'). PSII actual photochemical quantum yield or photosystem efficiency (phiPS2) and photosynthesis (P) followed a similar decline in the WSRI. Similarly, transpiration (E) and stomatal conductance (Gs) also demonstrated a similar decrease in the WSRI with waterlogging duration.

### 3.8 Towards simulation modeling of waterlogging effect on crop growth and development

Simulating the impact of waterlogging on cotton crop growth and development will assist in making informed decisions regarding cropping and management practices in regions exposed to waterlogging. This will also contribute to developing strategies for adapting to and coping with climate change. At the same time, it is intricate to develop a comprehensive model due to the complex dynamic interactions of waterlogging with the soil-plant atmospheric continuum (Liu et al., 2020b; Liu et al., 2023). For developing or improving a cotton model for waterlogging simulations, model conceptualization should be made from the point of view of soil and crop. The model would require simulating waterlogging-related parameters in the soil domain (in terms of spatial and temporally varying water table depth, water content, soil oxygen concentration, and surface ponding) and correlating these with crop growth and development-related parameters (primarilyphotosynthesis, growth, and development of plant organs, water,and nutrient uptake, and reproduction).

There are several studies on waterlogging, but only a few models (DRAINMOD (Skaggs et al., 2012), CROPR (Qian et al., 2017), SWAGMAN destiny (Meyer et al., 1996), AquaCrop (Steduto et al., 2009), WOFOST (De Wit et al., 2019), APSIM (Ebrahimi-Mollabashi et al., 2019)) have the capability of simulating the impact of waterlogging on crop growth and development (Liu et al., 2020b). These models can simulate the water table level fluctuations and associated stresses but lack process-based cotton crop modeling capabilities. In addition, the models also lack consideration of phenological delays caused by waterlogging (DRAINMOD, CROPR, AquaCrop, SWAGMAN, WOFOST), insufficient incorporation of plant adaptation and recovery mechanisms (DRAINMOD, AquaCrop, SWAGMAN, WOFOST), and the absence of crop growth stage-dependent effects of waterlogging (CROPR, AquaCrop, SWAGMAN, WOFOST) (Liu et al., 2020b). The existing cotton crop models (GOSSYM (Baker and McKinion, 1983; Beegum et al., 2023), OZCOT (Hearn, 1994), CSM-CROPGRO-Cotton (Hoogenboom et al., 1992), COTCO2 (Wall et al., 1994), COTTON2K (Marani, 2004)) cannot simulate the impact of waterlogging by accounting for the process based implications on cotton crop growth and development.

The conceptualization and methodology needed to incorporate the functional relationships developed in the current study will depend on the anticipated model type (simple empirical or mechanistic process-based model). In addition, if the aim is to improve an existing cotton model for waterlogging simulations, it is essential to link the soil and crop modeling concepts in those models with the functional relationships developed in this study. Accordingly, it is only possible to offer general remarks about developing a cotton crop model that can simulate the effects of waterlogging on cotton crops based on the findings of the present study.

Optimum conditions (solar radiation, temperature, atmospheric CO_2_, soil water, and nutrients) result in potential growth, yield, and biomass. A deviation from the optimum conditions results in variation in the potential plant growth capabilities (Nobel, 1984; Reddy et al., 2008). WSRI (**Figure 10**) and functional relationships established in this study for all shoot and root morphological parameters, plant pigments and gas exchange, photochemical, and fluorescence can be used to quantify the waterlogging stress effect on the potential cotton growth and development.

Effects of waterlogging on plant growth can be modeled by associating waterlogging impact on photosynthesis and net carbon assimilation. This can be established by using the functional relationship between photosynthesis and waterlogging duration (**Figures 4A**, **10D**). A similar approach is used in CROPR and SWAGMAN Destiny, in which the soil oxygen concentration represented in terms of drainage index/aeration stress factor is linked to the total dry matter in the crop (Meyer et al., 1996; Qian et al., 2017). In most of the existing cotton crop models, the carbon allocation to the plant’s organs is based on the carbon demand and carbon available. In GOSSYM model, which is one of the widely used cotton crop models, is represented in terms of carbon stress (ratio of carbon demand and carbon available) (Baker and McKinion, 1983; Hodges et al., 2018; Beegum et al., 2023). Therefore, a reduction in net photosynthesis or carbon availability as a function of waterlogging can be interrelated with the growth of plant organs. The present study observed that the relative response in dry weight of different plant organs is similar under different durations of waterlogging; therefore, it is not necessary to modifythe carbon allocation to different organs during waterlogging in theearly growth stage of cotton (**Figure 10B**). In addition, node formation/addition in the crop can also be correlated with carbon availability. For example, in GOSSYM model, node formation in the mainstem, vegetative branches and fruiting branches is a function of temperature, carbon, and nitrogen availability (Baker and Landivar, 1991).

Functional relationships developed from this study on the root growth and development parameters and waterlogging can be used to simulate root growth under waterlogged conditions (**Figure 6**, section 3.5). A similar approach is used in SWAGMAN density and APSIM soybean model, in which root growth and distribution are modeled as a function of aeration stress (Meyer et al., 1996; Ebrahimi-Mollabashi et al., 2019). The root morphological parameters should be associated with the water and nutrient uptake from the waterlogged soil. The influence of waterlogging on cotton plant height and leaf area can be established based on the separate functions developed in this study (**Figures 10A**, **Figures 7A, D**). Time to the first square is observed to be delayed with an increase in waterlogging duration (**Figure 9**). The functional relationship between the time to first square and waterlogging can be used to establish the delay. Since the first square is generally formed on nodes 5 to 7, the first square formation can also be correlated with the number of pre-fruiting nodes, whose formation can be a function of carbon availability associated with net photosynthesis reduction (Reddy, 1994).

This study is limited to the impact of waterlogging on the early growth stages of cotton. Waterlogging can have different effects depending on the growth stage of the cotton crop. Wang et al., 2017 observed that waterlogging at seedling, squaring, flowering, and boll opening reduced yield by 38.8%, 27.9%, 18.3%, and 7.6%, respectively (Wang et al., 2017). Future research is needed to develop functional relationships between waterlogging and plant growth and development parameters at higher growth stages (flowering and boll opening) to understand stage-dependent mechanisms under waterlogged conditions. Several previous studies have found that waterlogging significantly influences fiber quality (Wang et al., 2017). Therefore, the impact of waterlogging on fiber quality must also be investigated. Developing a robust, comprehensive model will depend on integrating functional relationships for all cotton growth stages and overall model conceptualization.

## 4 Conclusions

The present study analyzed the impact of different waterlogging durations on early cotton crop growth and development. Growth and development-related parameters were significantly affected by waterlogging. After waterlogging was initiated, the soil reached a hypoxic state, followed by an anoxic state. This resulted in a reduction in soil oxygen levels, affecting the roots’ functioning. Root growth was reduced, as evidenced by decreased root length, surface area, root volume, tips, and root crossings. This impacted the uptake of nutrients and resulted in a decrease in most of the micro/macronutrients and an increase in particular nutrients. This resulted in a nutrient deficiency and imbalance in the plant. As part of the plant’s protective mechanism in response to stress, stress-protective components (flavonoid, anthocyanin) increased. Reduced oxygen in the plant tissues and reduced/imbalance of plant nutrients impacted the photosynthesis and transpiration processes. This was apparent through the reduction in photosynthesis, transpiration, stomatal conductance, ETR, PhiPS2, Fv'/Fm', and an increase in NPQ. Reduced carbon assimilation in the plant led to a decrease in growth and development, as observed in the reduction in biomass of the plant organs, the number of nodes and leaves, and leaf area. Plant reproductive development was affected, as evidenced by the delay in the occurrence of the first square following the impact on growth and development. Based on the experiments, linear, exponential decay, and quadratic relationships and waterlogging stress response indices were established between the duration of waterlogging and cotton growth and development-related parameters. These can be used to develop or improve existing cotton models for simulating the impact of waterlogging.

## Data availability statement

The original contributions presented in the study are included in the article/**Supplementary Material**. Further inquiries can be directed to the corresponding authors.

## Author contributions

KR, RB, VT, VR, and DB contributed to conception and design of the study. KR, VT, and DB carried out the experiments. KR and SB organized the database. SB, KR, and RB performed the statistical analysis. SB wrote the first draft of the manuscript. RB, VR, and KR wrote sections of the manuscript. All authors contributed to the article and approved the submitted version.
