# Supplementary material for: Developing functional relationships between waterlogging and cotton growth and physiology-towards waterlogging modeling
Source: Front Plant Sci. 2023 Jul 31;14:1174682. doi: 10.3389/fpls.2023.1174682 (PMC10425224; doi:10.3389/fpls.2023.1174682)
Supplement: Supplementary file 1 [file Table_1.docx]

**Developing Functional Relationships Between Waterlogging and Cotton Growth and Physiology- Towards Waterlogging Modeling**

Sahila Beegum^ab*^, Van Truong^c^, Raju Bheemanahalli^c^, David Brand^c^, Vangimalla Reddy^a^ , and Kambham Raja Reddy^c*^

^a^Adaptive Cropping System Laboratory, USDA-ARS, Beltsville, MD, USA

^b^Nebraska Water Center, Robert B. Daugherty Water for Food Global Institute, University of Nebraska, Lincoln, NE, USA

^c^Department of Plant and Soil Sciences, Mississippi State University, Mississippi, MS, USA

*Corresponding authors; emails: [beegumsahila@gmail.com](mailto:beegumsahila@gmail.com) and [krreddy@pss.msstate.edu](mailto:krreddy@pss.msstate.edu)

**Supplementary file:**

**Table S1.** Summary of the analysis of variance across waterlogging treatment on various growth, development, and physiological traits measured 14 days after the start of the treatment; Plant height (PH, cm), number of leaves (LN, no plant^-1^), stem diameter (SD, cm), total leaf area (LA, cm^2^), leaf area per plant (LAP, cm^2^ plant^-1^), total leaf dry weight (TLDW, g plant^-1^), dry stem weight (StDW, g plant^-1^), root dry weight (RDW, g plant^-1^), shoot dry weight (ShDW, g plant^-1^), total plant dry weight (TPDW, g plant^-1^), root to shoot ratio (RSR, -), total root length (TRL, cm), root surface area (RSA, cm^2^), average root diameter (ARD, cm), root volume (RV, cm^3^), number of root tips(RT,-), number of root forks (RF,-), root crossing (RC, -), number of days to first square (DFSq,-). Leaf photosynthesis (A, umol m^-2^ s^-1^), stomatal conductance (Gs, mol m^-2^ s^-1^), ratio of intercellular and ambient CO_2_ concentrations (Ci/Ca, -), and transpiration (E, mmol m^-2^ s^-1^), photosynthetic electron transport rate (ETR, umol m^-2^ s^-1^), PSII actual photochemical quantum yield or photosystem efficiency (PhiCO_2_, µmol µmol^-1^), nonphotochemical chlorophyll fluorescence quenching (NPQ), minimal fluorescence of a light-adapted leaf that has momentarily been darkened (Fo), and PSII effective chlorophyll fluorescence (Fv'/Fm'), Chlorophyll (Chl, µg m^-2^), flavonoid index (Flav), anthocyanin (Anth), and nitrogen balance index (NBI). Macronutrients (nitrogen (N, %), calcium (Ca, %), potassium (K, %), magnesium (Mg, %), phosphorous (P, %), sulphur (S, %)) and micronutrients (boron (B, ppm), copper (Cu, ppm), iron (Fe, ppm), manganese (Mn, ppm), and zinc (Zn, ppm)) in the leaf, stem, and root. The lower-case letters within the columns denote statistically significant difference between treatments according to the Fisher's LSD test, ***, **, and NS indicates significance at p<0.001, p<0.05, and p<0.01, respectively.

| Treatment | PH | LN | | SD | LA | LAP | TLDW | StDW | RDW | ShDW | TPDW | RSR | DFSq |
| --- | --- | --- | --- | --- | --- | --- | --- | --- | --- | --- | --- | --- | --- |
|  | *** | *** | | ** | *** | *** | *** | *** | *** | *** | *** | *** | *** |
| 0 | 47.10a | 9.40a | 10.16a | | 1419.90a | 1559.56a | 10.30a | 6.89a | 2.05a | 19.24a | 21.28a | 0.11bc | 25.00c |
| 2 | 46.36a | 9.00a | 9.00b | | 1398.91a | 1533.73a | 10.95a | 7.39a | 2.07a | 20.41a | 22.48a | 0.10bcd | 24.80c |
| 4 | 35.46b | 7.80b | 6.42c | | 648.61b | 766.18b | 5.06b | 3.35b | 1.43b | 9.84b | 11.27b | 0.15a | 27.00b |
| 6 | 30.82c | 7.20b | 6.16c | | 431.14c | 515.91c | 3.64bc | 2.47bc | 1.01c | 7.12bc | 8.13bc | 0.15a | 29.00a |
| 8 | 25.06d | 6.20c | 5.04d | | 248.47cd | 305.04cd | 2.66c | 1.72c | 0.64d | 5.02c | 5.66cd | 0.13ab | 0.00d |
| 10 | 23.74d | 6.20c | 4.22de | | 233.43cd | 246.62d | 2.52c | 1.66c | 0.46d | 4.64c | 5.10cd | 0.10cd | 0.00d |
| 14 | 23.08d | 5.60c | 3.96e | | 195.22d | 278.84d | 2.79c | 1.81c | 0.37d | 4.97c | 5.34cd | 0.08d | 0.00d |
| 12 | 21.68d | 5.60c | 3.96e | | 163.98d | 206.27d | 2.30c | 1.59c | 0.33d | 4.22c | 4.56d | 0.08d | 0.00d |

| Treatment | TRL | RSA | ARD | RV | RT | RF | RC |
| --- | --- | --- | --- | --- | --- | --- | --- |
|  | *** | *** | *** | *** | *** | *** | *** |
| 0 | 8667.92a | 1307.29a | 1.05a | 14.67a | 9395.00a | 54040.20a | 4823.80a |
| 2 | 8832.87a | 1306.41a | 0.97a | 14.67a | 9885.80a | 57119.80a | 4418.20a |
| 4 | 7141.35ab | 1150.30ab | 0.91ab | 13.05a | 8682.00a | 38208.00b | 3379.00b |
| 6 | 6589.53b | 1017.72b | 0.78b | 11.66a | 6823.53b | 29116.40b | 2654.60b |
| 8 | 3898.46c | 589.57c | 0.58c | 7.10b | 4403.20c | 15831.40c | 1581.20c |
| 10 | 2421.47cd | 351.25cd | 0.46c | 4.08bc | 3292.20c | 8104.00cd | 975.20c |
| 12 | 1840.71d | 319.01d | 0.47c | 3.42c | 3549.20c | 5964.80cd | 676.00c |
| 14 | 1584.46d | 227.30d | 0.46c | 2.61c | 4362.60c | 5360.00d | 576.40c |

| Treat  ment | E | A | Ci\Ca | Gs | ETR | PhiCO2 | NPQ | Fo | Fv'/Fm' | Chl | Flav | Anth | NBI |
| --- | --- | --- | --- | --- | --- | --- | --- | --- | --- | --- | --- | --- | --- |
|  | *** | *** | *** | *** | *** | *** | *** | *** | *** | NS | *** | ** | *** |
| 0 | 10.7 b | 34.4bc | 0.78a | 0.7b | 295.6ab | 0.03bc | 0.29de | 730.7ab | 0.66ab | 27.6a | 1.5e | 0.16c | 18.1a |
| 2 | 13.8 a | 41.2a | 0.82a | 1.0a | 329.6a | 0.03a | 0.15e | 757.4a | 0.68a | 25.4a | 1.7cd | 0.18abc | 18.0a |
| 4 | 12.9 ab | 37.5ab | 0.81a | 0.9ab | 310.8ab | 0.03ab | 0.20de | 747.1a | 0.67a | 27.7a | 1.5e | 0.16c | 15.9ab |
| 6 | 11.1 b | 35.6abc | 0.80a | 0.8b | 295.7ab | 0.03abc | 0.30cde | 729.0ab | 0.66ab | 25.4a | 1.6de | 0.17bc | 15.0bc |
| 8 | 12.4 ab | 33.4bc | 0.83a | 0.9ab | 285.6abc | 0.03bc | 0.40cd | 708.9bc | 0.64bc | 26.1a | 1.7bc | 0.16c | 14.6bcd |
| 10 | 11.2 c | 31.8c | 0.81a | 0.8b | 272.6bc | 0.03c | 0.49c | 694.2c | 0.63c | 26.1a | 1.9a | 0.18abc | 13.7bcd |
| 12 | 3.6 c | 19.4d | 0.57b | 0.2c | 244.7c | 0.02d | 0.75b | 651.0d | 0.59d | 25.8a | 1.8ab | 0.19ab | 13.4cd |
| 14 | 1.7 c | 10.5e | 0.55b | 0.1c | 178.1d | 0.01e | 1.11a | 601.2e | 0.54e | 24.2a | 1.9a | 0.21a | 12.6d |

| **Leaf** | | | | | | | | | | | |
| --- | --- | --- | --- | --- | --- | --- | --- | --- | --- | --- | --- |
| **Treatment** | **Na** | **Ca** | **K** | **Mg** | **P** | **S** | **B** | **Cu** | **Fe** | **Mn** | **Zn** |
|  | *** | *** | *** | *** | *** | *** | NS | ** | ** | NS | ** |
| **0** | 3.56ab | 2.71a | 2.29ab | 0.46a | 0.35a | 0.5a | 39.79a | 0.74a | 106.6abc | 38.81b | 17.78a |
| **2** | 3.6a | 2.75a | 2.56ab | 0.49a | 0.36a | 0.38b | 42.81a | 0.38b | 149.3ab | 37.25b | 11.3b |
| **4** | 3.93a | 3.19a | 2.82a | 0.54a | 0.45a | 0.41b | 48.39a | 0.32b | 115.35abc | 49.78a | 11.8b |
| **6** | 3.82a | 2.84a | 2.51ab | 0.45a | 0.36a | 0.36b | 46.03a | 0.32b | 168.07a | 49.84a | 12.34b |
| **8** | 2.8b | 2.02b | 2.03b | 0.3b | 0.23b | 0.26c | 42.86a | 0.32b | 86.19bc | 42.89ab | 11.46b |
| **10** | 1.85c | 1.37bc | 1.45c | 0.22bc | 0.13b | 0.18d | 38.33a | 0.32b | 83.01bc | 43.64ab | 13.21ab |
| **12** | 1.64c | 1.19c | 1.27c | 0.19bc | 0.12b | 0.16d | 37.97a | 0.32b | 77.15c | 34.83b | 9.01b |
| **14** | 1.45c | 1.13c | 1.14c | 0.18c | 0.13b | 0.15d | 37.26a | 0.32b | 79.42bc | 36.3b | 10.1b |

| Root | | | | | | | | | | | |
| --- | --- | --- | --- | --- | --- | --- | --- | --- | --- | --- | --- |
| Treatment | **Na** | **Ca** | **K** | **Mg** | **P** | **S** | **B** | **Cu** | **Fe** | **Mn** | **Zn** |
|  | NS | NS | NS | NS | *** | NS | ** | *** | ** | NS | NS |
| 0 | 2.43de | 0.52ab | 3.13bc | 0.25abc | 0.36ab | 0.2abc | 43.54bc | 15.32d | 713.05cd | 81.1bc | 71.14b |
| 2 | 2.56bcde | 0.54ab | 2.74c | 0.28ab | 0.37ab | 0.17bc | 40.85c | 13.65d | 575.11d | 89.06abc | 33.43b |
| 4 | 3.16ab | 0.51ab | 4.06ab | 0.33ab | 0.39a | 0.23ab | 54.52abc | 14.59d | 943.68ab | 101.54a | 159.7a |
| 6 | 3.21a | 0.55ab | 4.57a | 0.38a | 0.36ab | 0.25a | 68.79a | 26.43bc | 720.65bcd | 84.44abc | 47.29b |
| 8 | 3.04abcd | 0.58a | 3.46abc | 0.36a | 0.33b | 0.22ab | 59.7abc | 21.91c | 923.52abc | 82.97abc | 50.58b |
| 10 | 3.05abc | 0.58a | 3.5abc | 0.35a | 0.32bc | 0.23ab | 60.86abc | 22.64c | 770.44abcd | 76.08c | 48.23b |
| 12 | 2.48cde | 0.52ab | 2.66c | 0.2bc | 0.26cd | 0.17bc | 65.73ab | 29.25ab | 976.22a | 97.82ab | 52.14b |
| 14 | 2.3e | 0.48b | 2.39c | 0.14c | 0.24d | 0.13c | 59.07abc | 34.09a | 870.09abc | 78.26c | 52.52b |

| **Stem** | | | | | | | | | | | |
| --- | --- | --- | --- | --- | --- | --- | --- | --- | --- | --- | --- |
| Treatment | Na | Ca | K | Mg | P | S | B | Cu | Fe | Mn | Zn |
|  | *** | *** | *** | *** | *** | *** | NS | ** | ** | NS | ** |
| **0** | 3.56ab | 2.71a | 2.29ab | 0.46a | 0.35a | 0.5a | 39.79a | 0.74a | 106.6abc | 38.81b | 17.78a |
| **2** | 3.6a | 2.75a | 2.56ab | 0.49a | 0.36a | 0.38b | 42.81a | 0.38b | 149.3ab | 37.25b | 11.3b |
| **4** | 3.93a | 3.19a | 2.82a | 0.54a | 0.45a | 0.41b | 48.39a | 0.32b | 115.35abc | 49.78a | 11.8b |
| **6** | 3.82a | 2.84a | 2.51ab | 0.45a | 0.36a | 0.36b | 46.03a | 0.32b | 168.07a | 49.84a | 12.34b |
| **8** | 2.8b | 2.02b | 2.03b | 0.3b | 0.23b | 0.26c | 42.86a | 0.32b | 86.19bc | 42.89ab | 11.46b |
| **10** | 1.85c | 1.37bc | 1.45c | 0.22bc | 0.13b | 0.18d | 38.33a | 0.32b | 83.01bc | 43.64ab | 13.21ab |
| **12** | 1.64c | 1.19c | 1.27c | 0.19bc | 0.12b | 0.16d | 37.97a | 0.32b | 77.15c | 34.83b | 9.01b |
| **14** | 1.45c | 1.13c | 1.14c | 0.18c | 0.13b | 0.15d | 37.26a | 0.32b | 79.42bc | 36.3b | 10.1b |
